# Effect and clinical significance of fast-track surgery combined with laparoscopic radical gastrectomy on the plasma level of vascular endothelial growth factor in gastric antrum cancer

**DOI:** 10.1186/s40064-016-1699-2

**Published:** 2016-01-20

**Authors:** Fa Fang, Jie Gao, Xing Bi, Feng Han, Hai-jiang Wang

**Affiliations:** Department of Gastrointestinal Surgery, The Tumor Hospital of Xinjiang Medical University, No. 789, Suzhou Street, Xinshi District, Ürümqi, 830000 Xinjiang China; Department of Medical Examination Center, The Tumor Hospital of Xinjiang Medical University, No. 789, Suzhou Street, Xinshi District, Ürümqi, 830000 Xinjiang China; Department of Urinary Surgery, The Tumor Hospital of Xinjiang Medical University, No. 789, Suzhou Street, Xinshi District, Ürümqi, 830000 Xinjiang China; Department of Emergency, The Tumor Hospital of Xinjiang Medical University, No. 789, Suzhou Street, Xinshi District, Ürümqi, 830000 Xinjiang China

**Keywords:** Gastric antrum cancer, Laparoscopy, Vascular endothelial growth factor, Fast-track surgery, Survival rate

## Abstract

This study discusses the effect and clinical significance of fast-track surgery (FTS) combined with laparoscopic radical surgery on the plasma level of vascular endothelial growth factor (VEGF) in locally advanced gastric antrum cancer. Plasma VEGF levels were detected in 63 cases of locally advanced gastric antrum cancer by using double-antibody sandwich Avidinbiotincomplex-ELISA before and after operation. The pure laparoscopic surgery group (group A) comprised 30 cases, and the combined FTS group (group B) consisted of 33 cases. Results of the two groups were obtained at similar time points and then compared. The VEGF levels were not significantly different between the two groups on the first day before the operation and on the first day, third day, and sixth month after the operation (P > 0.05). However, the differences were significant on the seventh day and first month after the operation (P < 0.05). The postoperative eating time, anal exhaust time, and hospital stay of the patients were statistically significantly different between the two groups (P < 0.05). Nevertheless, no significant differences were detected in terms of wound healing time and complications (P > 0.05). The 3-year survival rate significantly differed between the two groups (P < 0.05). FTS combined with laparoscopic surgery can decrease the postoperative VEGF level compared with pure laparoscopic surgery. The combined approach improved postoperative recovery without prolonging the wound healing time or increasing the incidence of postoperative complications. The 3-year survival rate also increased. Thus, FTS combined with laparoscopic surgery can improve the prognosis in gastric antrum cancer.

## Background

Gastric cancer is a prevalent malignant cancer worldwide and ranks second among all cancers in terms of mortality (Parkin et al. 2005). Surgery is the commonly used and the most effective approach for treatment of gastric cancer. In recent years, laparoscopic surgery has provided a new solution to gastric cancer treatment. Goh et al. (2001) first reported treatment of advanced gastric cancer by using laparoscopic D2 radical gastrectomy in 1997. The safety and feasibility of this approach have been recognized by many scholars.

Postoperative recovery involves the synergism of various factors. Wilmore et al. (Wilmore and Kehlet 2001; Kehlet and Wilmore 2002) proposed the concept of fast-track surgery (FTS) in 2001; FTS aims to adopt optimized measures for perioperative treatment by resorting to evidence-based medicine. FTS can facilitate postoperative recovery by reducing postoperative physical and psychological stresses. During recovery and repair of cells and tissues, vascular endothelial growth factor (VEGF) is an important factor that regulates angiogenesis. VEGF plays a crucial role in wound healing by inducing neoangiogenesis, promoting collagen deposition, and stimulating epithelization (Bao et al. 2009; Barrientos et al. 2008). VEGF also provides oxygen and nutrition for tumor growth, infiltration, and metastasis. Reports showed (Belizon et al. 2008) that laparoscopic surgery decreases the increasing amplitude of postoperative VEGF and the probability of recurrence. Nevertheless, whether FTS combined with laparoscopic surgery can inhibit the increasing postoperative VEGF level, facilitate postoperative recovery, and improve survival without increasing the incidence of complications or wound healing time remains debatable. To elucidate the effect of FTS, scholars have compared the outcome of patients with locally advanced gastric antrum cancer after pure laparoscopic surgery or laparoscopic surgery combined with FTS. Changes in the plasma VEGF levels are regularly monitored to assess the influence of FTS combined with laparoscopic surgery on postoperative VEGF levels and on the prognosis. Research results are reported in this article.

## Patients and methods

### General information

We recruited 63 cases of locally advanced gastric antrum cancer admitted to our hospital (31 males and 32 females, aged 46–73 years old) from January 2009 to October 2011. Of these cases, 56 patients were Han, and seven patients were minority. Approval was obtained from the hospital ethics committee, and informed consent was signed by all patients. The general information of the two groups of patients showed no statistically significant difference (P > 0.05, Table 1).

**Table 1:** General clinical data of the two groups of patients with gastric antrum cancer

Item	Group A	Group B	*P* value
Sex (male/female)	16/14	15/18	0.53
Age (years)	61.53 ± 12.37	61.12 ± 12.46	0.83
Ethnic (Han/minority)	27/3	29/4	0.79
Preoperative complication (yes/no)	13/17	14/19	0.94
Surgical approach (Billorth I/Billorth II)	12/18	17/16	0.36
Pathological type			
Mucinous adenocarcinoma, signet ring cell carcinoma and undifferentiated adenocarcinoma	11	13	0.82
Highly/moderately differentiated adenocarcinoma	19	20	
Type of tumor (protruded type/ulcerative type)	14/16	12/21	0.41

### Inclusion and exclusion criteria

Inclusion criteria: pathologically confirmed as adenocarcinoma before surgery; no distant metastasis found in imaging and laboratory examinations; no serious functional disorder of major organs with surgical tolerance; and belong to the T2 or T3 stage by preoperative clinical staging. Exclusion criteria: received neoadjuvant chemotherapy; presented multiple primary gastric cancers; found with distant metastasis through intraoperative examination; and presence of bleeding, obstruction, and perforation requiring emergency rescue.

### Laparoscopic surgery and FTS

All surgeries were performed by the same group of surgeons. Conventional five-incision method was used. Pneumoperitoneum was established with pressure ranging from 12 to 14 mmHg, and a 10-mm Trocar and a laparoscope was successively inserted. For all patients, (1) D2 radical gastrectomy was performed; (2) en bloc excision was performed along the paratumor tissues; (3) and tumor-free principle was followed. For the combined FTS group, additional treatment measures were administered. Preoperative education and nutrition support therapy were enhanced. The postoperative fasting time was shortened. The nutrition canal was indwelled by 20–30 cm from the distal end of the anastomotic stoma. Insulation was also conducted, and 5-hydroxytryptamine receptor antagonist was given to relieve nausea and vomiting. Patients also started eating and moving around at an earlier time. Sufficient analgesic measures were further provided. The following conventional recovery measures were administered in the control group: normal diet before operation, preoperative fasting for 6–8 h; only the gastric tube was indwelled; the patient was not allowed to eat until the first anal exhaust; and targeted analgesics were provided according to the need of patients.

### Statistical method

SPSS 13.0 software was used. Attribute data were analyzed using χ^2^ test, and variable data were expressed as X ± S. Pearson’s correlation was calculated to compare the differences in the VEGF levels. Repeated measure analysis of variance was applied to compare the VEGF levels obtained at the same time points. A test for spherical symmetry was also performed (if the spherical symmetry hypothesis was null, Greenhouse–Geisser correction was performed). Kaplan–Meier single-factor analysis was conducted to compare the cumulative survival rate and the average survival time within 1–3 years after the operation. The significance level was set as α = 0.05.

### VEGF detection

Double-antibody sandwich ABC-ELISA was conducted. A VEGF-ELISA assay kit was purchased from Jingmei Biotech Co., Ltd. The procedures were performed in accordance to the instruction manual. Peripheral blood samples were collected from the patients in the morning on the first day before the operation and on the first day, third day, seventh day, first month, and sixth month after the operation. The plasma VEGF levels in the two groups were detected and compared.

### Follow-up

From the day of the surgery to December 2014, 59 cases were followed up and four cases were lost. The follow-up rate was 93.65 % (59/63), and the median follow-up time was 37.1 months. For the cases lost to follow-up, examination results on the last follow-up were used for statistical calculation. Data of the patients completely lost to follow-up and patients died for reasons other than the tumor were treated as truncated data. Chest X-ray, abdominal B ultrasound, and tumor-related antigen detection were performed every month within 6 months after the operation. Fibrogastroscopy and CT examination were conducted every 4–6 months.

## Results

### Comparison of surgical conditions and postoperative recovery

All patients successfully underwent laparoscopic surgery. The postoperative hospital stay was shortened in the combined FTS group compared with that in the pure laparoscopic group (18.5 ± 5.5 vs. 11.0 ± 2.0); the first anal exhaust time (4.0 ± 1.0 vs. 2.5 ± 0.5) and the postoperative eating time were statistically significantly earlier than those in the pure laparoscopic group (P < 0.05). No significant differences were further observed in terms of operation time, intraoperative bleeding volume, perioperative blood transfusion, postoperative complications, wound healing time, postoperative T staging and postoperative N staging (P > 0.05). In the pure laparoscopic group (group A), one case presented pulmonary infection, and one case presented with delayed gastric emptying. In the combined FTS group, one case demonstrated urinary tract infection and one case with subcutaneous emphysema. The two groups did not significantly differ in terms of the incidence of complications (P > 0.05, Table 2).

**Table 2:** Postoperative conditions of the two groups of patients

Item	Group A	Group B	*P* value
Operation time (min)	253.20 ± 85.09	252.91 ± 84.92	1.00
Intraoperative bleeding volume (ml)^a^	211	205	1.00
Perioperative blood transfusion (case)	1/29	1/32	0.95
Time of first anal exhaust (days)	4.0 ± 1.0	2.5 ± 0.5	0.03
Time for wound healing (days)	10.0 ± 1.0	11.5 ± 1.5	0.29
Postoperative eating time (days)	4.5 ± 1.0	2.0 ± 0.5	0.02
Postoperative complication (yes/no)	2/28	2/31	0.92
Postoperative hospital stay (days)	18.5 ± 5.5	11.0 ± 2.0	0.00
pT_1–2_/pT_3_	9/21	11/22	0.78
pN_0–1_/pN_2_	14/16	18/15	0.53

^a^Median for skewedly distributed data

### Comparison of plasma VEGF

On the first day before the operation and on the first day, third day, sixth day, and sixth month after the operation, the VEGF levels were not significantly different between the two groups (P > 0.05). Statistically significant differences were detected on the seventh day and first month after the operation (P < 0.05, Table 3).

**Table 3:** Comparison of VEGF levels of the two groups before and after operation (X ± S) pg/ml

Item	Group A	Group B	*P* value
Day 1 before operation	47.27 ± 34.17	47.51 ± 33.76	1.00
Day 1 after operation	54.38 ± 24.81	53.27 ± 23.96	0.94
Day 3 after operation	62.73 ± 25.13	59.18 ± 24.89	0.86
Day 7 after operation	79.93 ± 27.36	64.36 ± 28.14	0.04
1 month after operation	47.28 ± 28.15	36.67 ± 25.12	0.03
6 months after operation	36.32 ± 25.31	35.4 8 ± 24.95	0.85

### Postoperative follow-up

**Fig. 1:**
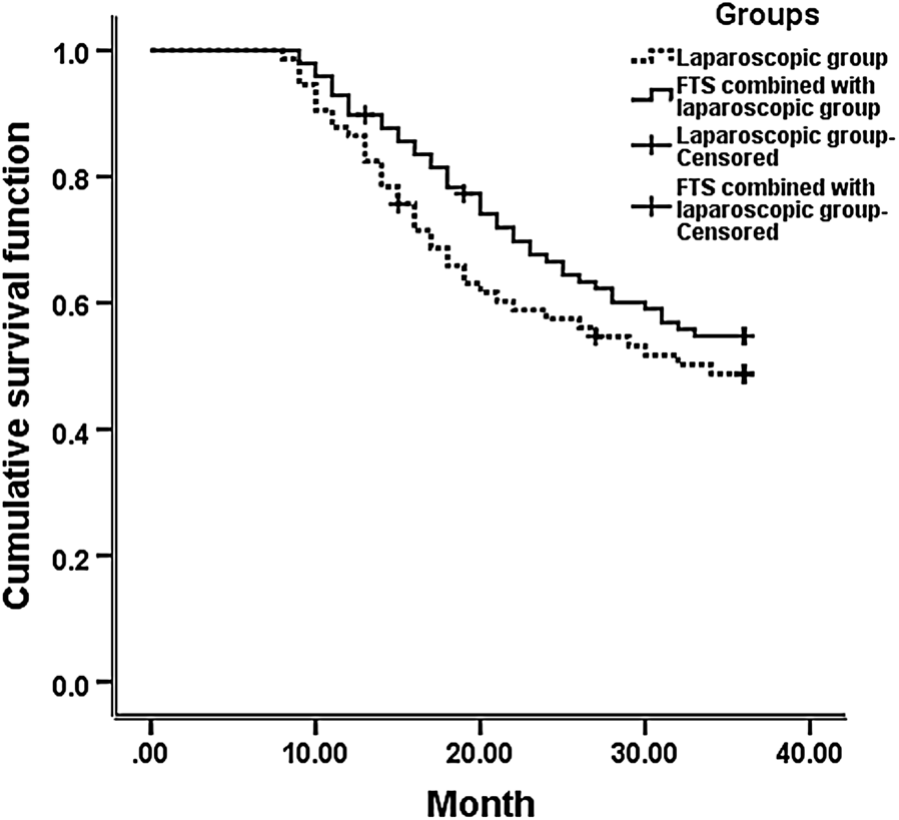
Three-year survival curves of the two groups

All patients underwent postoperative XELOX scheme chemotherapy 6 cycles. Kaplan–Meier analysis revealed that the 1-year cumulative survival rate was not statistically significantly different between groups A (86.60 %) and B (89.10 %) (P > 0.05). Nevertheless, the 3-year cumulative survival rate was significantly differed between groups A (48.30 %) and B (54.90 %) (P < 0.05). Moreover, the median survival time was statistically significantly different between groups A (26.11 months) and B (30.59 months) (P < 0.05, Fig. 1).

## Discussion

Surgeons primarily aim to reduce surgical trauma and facilitate postoperative recovery. Minimally invasive surgery can effectively reduce surgical trauma and intraoperative bleeding and produce smaller wounds, resulting in fast postoperative recovery. Nevertheless, less surgical trauma is insufficient, and scholars also focus on facilitating postoperative recovery. Currently, many proven measures are used preoperatively, intraoperatively, and postoperatively to reduce stress responses and relevant complications, thus improving the perioperative condition of patients. The combination of various therapies exhibits high effectiveness in relieving pain and facilitating postoperative recovery. In this study, the following additional measures were performed in the combined FTS group: preoperative education and nutrition support therapy were enhanced; the postoperative fasting time was shortened; the nutrition canal was indwelled by 20–30 cm from the distal end of the anastomotic stoma; insulation was conducted, and 5-hydroxytryptamine receptor antagonist was given to relieve nausea and vomiting; the patients started eating and moving around at an earlier time; and sufficient analgesic measures were administered. The results showed that patients in the combined FTS group presented a shortened postoperative hospital stay and earlier time of the first anal exhaust and postoperative eating. The differences were statistically significant (P < 0.05), which is consistent with previous reports (Soop et al. 2004; Correia and da Silva 2004; Lewis et al. 2001). FTS combined with laparoscopic radical surgery for treatment of gastric antrum cancer further relieved the stress responses, promoted the postoperative gastrointestinal recovery, and shortened the hospital stay.

VEGF is the most effective angiogenic factor that has been discovered. VEGF promotes the proliferation of endothelial cells and vascular leakage. VEGF also specifically induces the formation of lymphatic vessels and promotes collagen deposition and epithelization. Therefore, VEGF plays an important role in wound healing (Kigure et al. 2013; Wang et al. 2013). Organisms produce stress responses after trauma, leading to increased plasma VEGF levels, which play a role in wound healing and in reducing the leakage of anastomotic stoma. These postoperative stress responses can be effectively mitigated by FTS through decreasing the VEGF level. This research aimed to determine whether FTS affects wound healing and increases the incidence of complications. Our results indicated that the VEGF levels of patients in the two groups did not show significant difference on the first day before the operation and on the first day, third day, and sixth month after the operation (P > 0.05). On the seventh day and first month after the operation, the VEGF level in the combined FTS group decreased compared with that in the pure laparoscopic surgery group (P < 0.05). No differences were observed in the wound healing time and incidence of complications between the two groups (P > 0.05). Furthermore, the VEGF level in the combined FTS group was lower than that in the pure laparoscopic surgery group. FTS combined with laparoscopic surgery can facilitate postoperative recovery without affecting wound healing and postoperative complications in gastric antrum cancer. These results are consistent with those reported by Lordache et al. (2010).

Survival rate is an important indicator to evaluate the efficacy of therapies for malignant tumors and has also been an intensively researched topic. During the course of gastric cancer progression, lymph node metastasis is among the earliest signs of tumor cell dissemination. By promoting the proliferation of vascular endothelial cells and enhancing vascular permeability, VEGF induces tumor angiogenesis and the formation of lymphatic vessels. Thus, VEGF plays a crucial role in the growth and metastasis of gastric cancer (Wu et al. 2013; Feng et al. 2011). In surgical treatment of gastric cancer, the growth and spread of tumor cells can be inhibited by decreasing VEGF expression; nevertheless, the positive effect of this phenomenon remains debatable in terms of the recurrence, growth, and metastasis of gastric cancer and in decreasing the mortality by relieving stress responses and trauma. In this study, FST combined with laparoscopic radical surgery was used to treat gastric antrum cancer. The changes in the plasma VEGF level were monitored in locally advanced gastric cancer and then compared with the results obtained from pure laparoscopic surgery. The two groups showed an increasing VEGF level after operation until VEGF reached the peak. Subsequently, VEGF level decreased. The VEGF level increased with low amplitudes in the combined FTS group compared with that in the pure laparoscopic surgery group. The differences were statistically significant on the seventh day and first month after the operation (P < 0.05). Kaplan–Meier analysis showed that the 1-year cumulative survival rate was not significantly different between groups A (86.60 %) and B (89.10 %) (P > 0.05). Moreover, the 3-year cumulative survival rate was significantly different between groups A (48.30 %) and B (54.90 %) (P < 0.05). The median survival time was also significantly different between groups A (26.11 months) and B (30.59 months) (P < 0.05). The 3-year survival rates of patients with gastric antrum cancer increased by decreasing the VEGF level, which is consistent with previous reports (Weich et al. 2004; Tsutsumi et al. 2005; Al-Moundhri et al. 2008; He et al. 2008). Hence, the plasma VEGF level in gastric antrum cancer is significantly related to its prognosis. The VEGF level can be used as a suitable predictor of prognosis and as a reference to develop treatment regimens for gastric cancer.

In conclusion, FTS combined with laparoscopic radical surgery for gastric antrum cancer can effectively reduce stress responses and postoperative VEGF levels through the following measures: enhancing preoperative education and nutrition support therapy, shortening the time of preoperative fasting, indwelling the nutrition canal, sufficient insulation and analgesic treatment, earlier postoperative eating and moving around, relieving the psychological burden, and improving the postoperative gastrointestinal function and nutrition state.
